# The Therapeutic Effect of Curcumin in Quinolinic Acid-Induced Neurotoxicity in Rats is Associated with BDNF, ERK1/2, Nrf2, and Antioxidant Enzymes

**DOI:** 10.3390/antiox8090388

**Published:** 2019-09-11

**Authors:** Ricardo A. Santana-Martínez, Carlos A. Silva-Islas, Yessica Y. Fernández-Orihuela, Diana Barrera-Oviedo, José Pedraza-Chaverri, Rogelio Hernández-Pando, Perla D. Maldonado

**Affiliations:** 1 Laboratorio de Patología Vascular Cerebral, Instituto Nacional de Neurología y Neurocirugía, Insurgentes sur 3877, La Fama, Tlalpan CDMX 14269, Mexico; ricartiani@hotmail.com (R.A.S.-M.); yessicayaelfdz@gmail.com (Y.Y.F.-O.); 2 Departamento de Farmacología, Facultad de Medicina, Universidad Nacional Autónoma de Mexico, Av Universidad 3000, Coyoacán CDMX 04510, Mexico; dianabarrera@hotmail.com; 3 Departamento de Biología, Facultad de Química, Universidad Nacional Autónoma de Mexico, Av Universidad 3000, Coyoacán CDMX 04510, Mexico; pedraza@unam.mx; 4 Laboratorio de Patología Experimental, Instituto Nacional de Ciencias Médicas y Nutrición, Vasco de Quiroga, Belisario Dominguez 15, Secc 16, Tlalpan CDMX 1408, Mexico; rhdezpando@hotmail.com

**Keywords:** curcumin, BDNF, ERK1/2, Nrf2, glutathione, brain, quinolinic acid

## Abstract

In the present study we investigated the participation of brain-derived neurotropic factor (BDNF) on the activation of the mitogen activated protein kinase (MAPK) protein extracellular signal-regulated kinase-1/2 (ERK1/2) as a mechanism of curcumin (CUR) to provide an antioxidant defense system mediated by the nuclear factor erythroid 2-related factor 2 (Nrf2) in the neurotoxic model induced by quinolinic acid (QUIN). Wistar rats received CUR (400 mg/kg, intragastrically) for 6 days after intrastriatal injection with QUIN (240 nmol). CUR improved the motor deficit and morphological alterations induced by QUIN and restored BDNF, ERK1/2, and Nrf2 levels. CUR treatment avoided the decrease in the protein levels of glutathione peroxidase (GPx), glutathione reductase (GR), γ-glutamylcysteine ligase (γ-GCL), and glutathione (GSH) levels. Only, the QUIN-induced decrease in the GR activity was prevented by CUR treatment. Finally, QUIN increased superoxide dismutase 2 (SOD2) and catalase (CAT) levels, and the γGCL and CAT activities; however, this increase was major in the QUIN+CUR group for γ-GCL, CAT, and SOD activities. These data suggest that the therapeutic effect of CUR could involve BDNF action on the activation of ERK1/2 to induce increased levels of protein and enzyme activity of antioxidant proteins regulated by Nrf2 and GSH levels.

## 1. Introduction

Quinolinic acid (QUIN) is a neuroactive metabolite of the kynurenine pathway in the brain, which is the main route of catabolism of tryptophan to produce nicotinamide adenine dinucleotide (NAD^+^). QUIN is present in human and rat brains at nanomolar concentrations [1]; however, an increase in QUIN levels is observed in several neurodegenerative diseases [2]. There are reports indicating that the intrastriatal administration of QUIN can be used as a biochemical model to study the excitotoxicity and oxidative stress, two mechanisms involved in the pathophysiology of neurodegenerative diseases. Particularly, QUIN administration reproduces some biochemical, histopathological, and behavioral alterations observed in Huntington disease [3] because selectively activates the N-methyl-D-aspartate receptors containing GluN2A and GluN2B subunits [4], which are highly expressed in the striatum (involved in motor control, learning, language, reward, and cognitive functioning), hippocampus, and cortex in the adult human and rat brain [5,6]. QUIN administration also stimulates superoxide anion [7] and hydroxyl radical production [8], leading to oxidative damage that is implicated in the pathophysiology of neurodegenerative diseases [9].

Cells have an adaptive response system to endure oxidative stress; these include a battery of cytoprotective genes involved in (1) the detoxification of xenobiotics (glutathione S-transferase, GST); (2) the regulation of reduced nicotinamide adenine dinucleotide phosphate (NADPH) levels (glucose 6-phosphate dehydrogenase; G6PDH); (3) the homeostasis of glutathione (GSH) levels (γ-glutamylcysteine ligase, γ-GCL; glutathione peroxidase, GPx; and glutathione reductase, GR); (4) the decrease of oxidative stress (catalase, CAT; and superoxide dismutase 1 and 2, SOD1 and SOD2), and other processes [10]. The main regulator of these genes is the nuclear factor erythroid 2-related factor 2 (Nrf2) [11]. Nrf2 is involved in the regulation of the transcription of cytoprotective genes through its binding to the antioxidant response element (ARE), present in the upstream promoter region of more than 600 genes [10]. Nrf2 activation occurs through the oxidation of specific reactive cysteine residues of the Kelch-like erythroide cell-derived protein with cap’n’collar homology (ECH)-associated protein 1 (Keap1), the cytoplasmic repressor of Nrf2, resulting in its accumulation, nuclear translocation, and activation [12]. Since Nrf2 is rapidly degraded by the 26S proteasome (half-life ≈ 15 min), strategies to keep optimal levels of this factor are necessary.

There is evidence indicating that the Nrf2 stabilization in vitro can occur via the activation of mitogen activated protein kinases (MAPK) such as extracellular signal-regulated kinase-1/2 (ERK1/2) and c-Jun-amino (NH) terminal-kinase (JNK) [13]. ERK1/2 is a kinase implicated in cell adhesion, cell cycle progression, and cell migration and survival [14]. Its phosphorylation (activation) mainly occurs as a result of growth factor/cytokine stimulation [15]; however, it also occurs due to oxidizing stimuli such as hydrogen peroxide (H_2_O_2_) [16].

A variety of naturally occurring compounds can stimulate Nrf2 activation, such as curcumin (CUR, 1,7-bis (4-hydroxy-3-methoxyphenyl)-1,6-hetadiene-3,5-dione) [17], a hydrophobic polyphenol derived from the rhizome of the herb *Curcuma longa* Linn [18]. There are reports demonstrating the cytoprotective properties of CUR as an antioxidant in several preclinical models of Huntington [19], Alzheimer [20], and Parkinson [21] diseases. CUR acts as both a free radical scavenger (direct antioxidant) [22] and as an Nrf2 inducer (indirect antioxidant) [23], although the mechanism by which CUR activates Nrf2 remains unclear. Recently, Bucolo et al. [24] demonstrated that CUR protects human retinal pigment epithelial cells against high glucose toxicity through the Nrf2 activation mediated by ERK1/2 phosphorylation. Additionally, a CUR analogue (bisdemethoxycurcumin) induces the phosphorylation of ERK1/2 in a Ca^2+^/calmodulin-dependent protein kinase II (CaMKII)-dependent manner, enhancing the expression of heme oxygenase 1, a protein regulated by Nrf2 [25]. Moreover, CUR is able to activate the brain-derived neurotropic factor (BDNF) signaling pathway and confer protection in an in vivo model of neurodegeneration induced by alcohol and arsenic in the hippocampus [26] and striatum [27]. BDNF is a member of the neurotrophin family and plays a crucial role in the maintenance of adult neuronal function [28,29]. The binding of BDNF to its target receptor, the tropomyosin receptor kinase-B (TrkBr), which triggers the activation of phosphatidylinositol 3-kinases (PI3K) and ERK1/2 signaling pathways [30]. A study indicates that a cross-talk between PI3K and ERK1/2 signaling activated by BDNF may play a prominent role in the preservation of dopaminergic function in the striatum [31]. Considering the previous evidence, we hypothesized that the accumulation of Nrf2 induced by CUR could be related with activation of ERK1/2 in a manner dependent on the BNDF signaling pathway in the QUIN model.

## 2. Materials and Methods

### 2.1. Chemicals

QUIN, CUR, o-ophthaldehyde (OPA), NADPH, β-nicotinamide adenine dinucleotide phosphate (NADP^+^), GR, GSH, oxidized glutathione (GSSG), 2,3-naphthalenedicarboxyaldehyde (NDA), H_2_O_2_, 1-choloro-2,4-dinitrobenzene (CDNB), glucose 6-phosphate, dithiothreitol, bovine serum albumin (BSA), ethylenediamine tetraacetic acid (EDTA), paraformaldehyde (PAF), phenylmethylsulfonyl fluoride (PMSF), protease and phosphatase inhibitors, and primary antibody anti-α-tubulin were obtained from Sigma-Aldrich (St. Louis, MO, USA). Phosphoric acid (H_3_PO_4_) was obtained from Golden Bell Reagent (Guadalajara, Jalisco, Mexico). Fluoro-Jade B (FJ-B) and polyvinylidene fluoride (PVDF) membrane were obtained from Millipore (Bedford, MA, USA). Primary antibodies anti-Nrf2 (C-20), anti-GR, anti-γ-GCLc, anti-CAT, anti-phospho-ERK1/2, and anti-ERK1/2 were obtained from Santa Cruz Biotechnology Inc. (Dallas, TX, USA). Primary antibodies anti-BDNF and anti-GPx were obtained from Abcam (Cambridge, MA, USA). Primary antibodies anti-SOD1 and anti-SOD2 were obtained from Enzo Life Science (Farmingdale, NY, USA). Donkey anti-rabbit, anti-mouse, and anti-goat horseradish peroxidase-conjugate antibodies (secondary antibodies) were from Jackson Immunoresearch Laboratories Inc. (West Grove, PA, USA). Deionized water from a Milli-Q system (Millipore) was used for preparation of solutions.

### 2.2. Animals

Male Wistar rats (280–320 g) were housed five per cage in acrylic box cages and provided with a standard commercial rat chow diet (Laboratory rodent diet 5001; PMI Feeds Inc., Richmond, IN, USA) and water ad libitum. The housing room was maintained under constant conditions of temperature (25 ± 3 °C), humidity (50 ± 10%), and lighting (12-h light/dark cycles). All experimental procedures with animals were carried out strictly according to the National Institutes of Health Guides for the Care and Use of Laboratory Animals and the Local Guidelines on the Ethical Use of Animals from the Health Ministry of Mexico (NOM-062-ZOO-1999) and were approved by the Local Ethics Committee of Instituto Nacional de Neurología y Neurocirugía (INNN 44/15 project approved at 15 September 2015). All efforts were made to minimize animal suffering.

### 2.3. Experimental Design

**Figure 1:**
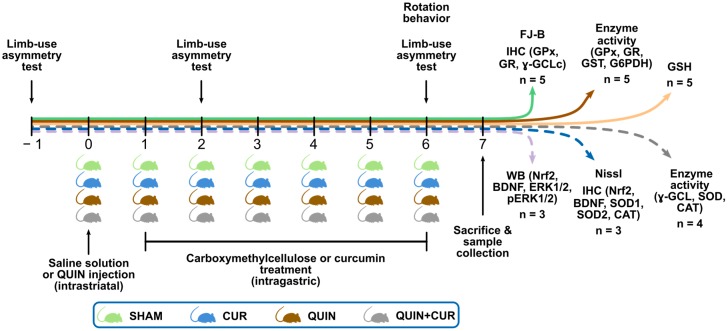
Experimental design. Animals were divided into four groups (day 0): SHAM, Curcumin (CUR), quinolinic acid (QUIN), and QUIN plus CUR (QUIN+CUR), and injected in the right striatum with 1 µL of saline solution (SHAM and CUR groups) or 240 nmol QUIN (QUIN and QUIN+CUR groups). Twenty-four hours after the saline solution or QUIN injections (day −1), animals were intragastrically administered with six doses (every 24 h) of carboxymethylcelullose (SHAM and QUIN groups) or CUR (400 mg/kg) (CUR and QUIN+CUR groups). The limb-use asymmetry test was evaluated 24 h before the saline solution or QUIN administration (day −1) and on days 2 and 6. The rotation behavior was evaluated on day 6. Horizontal solid arrows in color indicate groups of animals subjected to motor assessment. Six independents groups of animals (indicated with the horizontal arrows in color) were sacrificed (day 7) and the striatum or brains were obtained to perform the indicated assays. The number of animals (n) used in each independent group is indicated. FJ-B: fluorojade-B, GPx: glutathione peroxidase, GR: glutathione reductase, γ-GCLc: catalytic subunit of γ-glutamylcysteine ligase, GST: glutathione S-transferase, G6PDH: glucose 6 phosphate dehydrogenase, SOD1: superoxide dismutase 1, SOD2: superoxide dismutase 2, CAT: catalase, GSH: reduced glutathione, Nrf2: nuclear factor erythroid 2-related factor 2, BDNF: brain-derived neurotropic factor, ERK1/2: mitogen activated protein kinase and extracellular signal-regulated kinase-1/2, p-ERK1/2: phosphorylated ERK1/2, IHC: immunohistochemistry, WB: Western blot.

Animals were randomly divided into four groups (*n* = 3–5) as follows: (1) SHAM group rats were intrastriatally injected with saline solution and treated with carboxymethylcellulose (intragastrically: i.g.); (2) CUR group rats were intrastriatally injected with saline solution and treated with CUR (i.g.); (3) QUIN group rats were instrastriatally injected with QUIN and treated with carboxymethylcellulose (i.g.); and (4) QUIN+CUR group rats were instrastriatally injected with QUIN and treated with CUR (i.g.). Animals in the CUR and QUIN+CUR groups received a daily dose of CUR in 0.5% carboxymethylcellulose (400 mg/kg, i.g.), for 6 consecutive days. The first dose of CUR was administered 24 h after the striatal injection of the saline solution or QUIN. Twenty-four hours after the last CUR dose (day 7) the rats were sacrificed. Six independents groups of animals (indicated with the horizontal arrows in color) were used to evaluate: (1) the neuronal degeneration using fluorojade-B (FJ-B) staining and the levels of GPx, GR, and catalytic subunit of γ-GCL (γ-GCLc) using immunohistochemistry (IHC); (2) the enzyme activity of GPx, GR, GST, and G6PDH; (3) the GSH levels; (4) the histological damage using Nissl staining and the levels of Nrf2, BDNF, SOD1, SOD2, and CAT using IHC; (5) the total levels of Nrf2, BDNF, ERK1/2, and p-ERK1/2 uisng Western blotting; and (6) the enzyme activity of γ-GCL, SOD, and CAT. The limb-use asymmetry test was evaluated 24 h before the saline solution or QUIN administration (day −1) and on days 2 and 6. The rotation behavior was evaluated on day 6 (Figure 1).

### 2.4. Striatal Lesion

Rats were anesthetized with sodium pentobarbital (50 mg/kg, intraperitoneally (i.p.)) and received an injection of 1 µL of saline solution or QUIN (240 µmol) with a Hamilton microsyringe into the right striatum using the following stereotaxic coordinates: +0.5 mm anterior to bregma, −2.6 mm lateral to bregma, and −4.5 mm ventral to dura [32]. QUIN was dissolved in 0.9% isotonic saline solution (saline solution) and the pH was adjusted to 7.4 with NaOH.

### 2.5. Motor Assessment

#### 2.5.1. Limb-Use Asymmetry Test

The limb-use asymmetry test was assessed according to a previous report with some modifications [33]. The test was performed one day before the lesion (day − 1) as well as on days 2 and 6 after the saline solution or QUIN injection. All evaluations were performed at 22:00 h. Rats were placed in a clear Plexiglas cylinder (30 cm tall by 20 cm diameter) for 5 min. The number of contacts on the wall using forelimbs, ipsilateral forelimb (side of the lesion), and contralateral forelimb (opposite side of the lesion) was quantified. The forelimb use percentage was obtained by multiplying the number of contacts using forelimbs by 100 and divided it by total number of contacts (contacts with forelimbs, ipsilateral forelimb plus contralateral forelimb). The asymmetry score was calculated by adding ipsilateral forelimb contact plus half the number of contacts with forelimbs, divided by the total number of contacts (ipsilateral forelimb, contralateral limb plus forelimbs contacts). This test provides an asymmetry score; a score >0.5 indicates a greater reliance on the ipsilateral forelimb, whereas a score <0.5 indicates a greater reliance on the contralateral forelimb.

#### 2.5.2. Behavioral Test

Rotation behavior was quantified according to a previous report [7]. Six days after the saline solution or QUIN injection, animals from all groups were administered with apomorphine (1 mg/kg, subcutaneously (s.c.))s.c.) and separated into individual acrylic box cages. Five minutes later, the number of ipsilateral rotations was recorded for 1 h. Each rotation was defined as a complete 360° turn. Data were expressed as the total numbers of turns in 1 h.

### 2.6. Histological and Immunohistochemical Analyses

#### 2.6.1. Obtaining and Treatment of Samples to Histological Analysis

Brains were collected and histologically processed according to previous descriptions [34]. Seven days after the saline solution or QUIN injection, rats were anesthetized with sodium pentobarbital (200 mg/kg, i.p.) and perfused transcardially with saline solution plus heparin (1/500, *v*/*v*), followed by cold 4% PAF (*v*/*v*) solution. Brains were removed and postfixed in 4% PAF for 48 h. Subsequently, the brains were gradually dehydrated in absolute ethanol followed by xylene and immersed in Paraplast Plus parafine (McCormick Scientific, St. Louis, MO, USA). Tissues were serially sectioned in an 820 Histo-STAT microtome (American Instrument Exchange, Inc., Haverhill, MA, USA). Coronal sections (5 µm) were obtained every 100 µm, covering a total distance of 300 µm (100 µm anterior and 100 µm posterior to the needle tract). The quantification of the cells positive to Nissl and FJ-B was performed in three randomly selected fields along the right striatum.

#### 2.6.2. Nissl Staining

Nissl staining was performed according to a previous study [34]. Coronal sections (5 µm) were deparaffinized and collocated in absolute ethanol, washed with tap water, and stained with 0.1% cresyl violet for 1 min. Slides were rinsed with distilled water, dehydrated with absolute alcohol and xylene, and mounted with DPX mounting medium. Images of the striatum from each animal were obtained with a Nikon E 200 microscope (Nikon, Melville, NY, USA) using a 40× objective. Results were expressed as the percentage of cells positive to Nissl per field. A positive cell of Nissl is defined as a cell that contain a Nissl body, a large granular body of rough endoplasmic reticulum with rosettes of free ribosomes.

#### 2.6.3. Fluoro-Jade B (FJ-B) Staining

FJ-B staining is a sensitive marker of neurons undergoing degeneration [35]. Coronal sections (8 µm) were immersed in xylene for 10 min, followed by absolute ethanol for 2 min, 1% NaOH–80% ethanol solution for 5 min, and finally in 70% ethanol. Subsequently, slides were immersed in a solution of 0.06% KMnO_4_ for 10 min with moderate shaking and were rinsed with distilled water for 2 min. Slides were stained with 0.0004% FJ-B solution for 20 min and were washed thrice with distilled water. Excess water was removed by placing the slides in an oven at 50 °C for 15 min. Finally, the slices were placed in xylene and covered with non-aqueous mounting medium. Images of the striatum from each animal were obtained with a Nikon E 200 microscope (Nikon, Melville, NY, USA) using a 40× objective. Results are expressed as the number of cells positive to FJ-B per field.

#### 2.6.4. Immunohistochemistry

Coronal sections (8 µm) were immersed in 10 mM sodium citrate pH 6.0 plus 0.2% Triton X-100 for 15 min. Endogenous peroxidase activity was inactivated with 1% H_2_O_2_. Slides were blocked with 0.01% BSA for 1 h and then were incubated with the primary polyclonal antibodies anti-BDNF (1:100), anti-Nrf2 (1:50), anti-GPx (1:200), anti-GR (1:200), anti-γ-GCLc (1:200), anti-SOD1 (1:100), anti SOD2 (1:250), and anti-CAT (1:50) for 24 h. Slides were incubated with a Universal L Kit SAB-System HRP (secondary antibody and peroxidase; Dako North America Inc., Carpinteria, CA, USA). Subsequently, slides were developed with 3,3′-diaminobenzidine and were slightly counterstained with hematoxylin. Finally, the slices were covered with non-aqueous mounting medium. Images of three randomly selected fields along the right striatum from each animal were obtained with a Nikon E 200 microscope (Nikon, Melville, NY, USA) using a 40× objective. Results were expressed as the number of positive cells per field.

### 2.7. Preparation of Total Homogenates Used for Enzyme Activity and Western Blot Assays

Striatal samples were homogenized in 500 μL of cold lysis buffer pH 7.9 (20 mM Tris HCl, 30 mM NaCl, 0.5 mM sucrose, 1 μg/μL leupeptin, 1 μg/μL aprotinin, 1 μg/μL pepstatin, and 1 μM PMSF), and centrifuged at 20,800× *g* for 30 min at 4 °C. The supernatants were used to determine the enzyme activity of GPx, GR, GST, G6PDH, γ-GCL, SOD, and CAT, and for Western blot assays. Protein content was determined using Lowry’s method.

### 2.8. Western Blot Assay

Fifty micrograms of total protein of whole lysate were loaded and separated in 12% sodium dodecyl sulfate-polyacrylamide gel electrophoresis, and further transferred to PVDF membranes. Membranes were blocked with 5% nonfat milk for 2 h at room temperature with moderate agitation. Blots were incubated with primary antibodies anti-Nrf2 (1:1,000), anti-BDNF (1:1000), anti-phospho-ERK1/2 (1:1000), anti-ERK1/2 (1:1000), or anti-α-tubulin (1:8000) overnight at room temperature. Membranes were washed three times (10 min each) with 20 mM Tris, 0.5 M NaCl, and 0.1% Tween^®^. Horseradish peroxidase-conjugated secondary antibodies anti-rabbit (1:10,000), anti-mouse (1:10,000), and anti-goat (1:10,000) were added for 2 h, followed by an extensive washing. Bands were detected using an enhanced chemiluminescence detection system Immobilon® Western (Millipore Corporation, Billerica, MA, USA) with a photodocumenter (Vilber Lourmat, Eberhardzell, Germany). Area values of each group were obtained and then standardized to the area value of the SHAM group (value = 1). Data were expressed as the relative optical density (O.D.) of the protein/α-tubulin (ImageJ v1.52a, NIH, Bethesda, MD, USA).

### 2.9. Activity of Antioxidant Enzymes

GCL activity was measured using a previously described spectrophotometric method [36]. Twenty microliters of samples were added to 50 µL of the reaction mix (400 mM Tris-base, 20 mM L-glutamic acid, 2 mM EDTA, 20 mM boric acid, 2 mM L-serine, 10 mM MgCl_2_, and 40 mM ATP). The reaction was initiated by the addition of 50 µL of 2 mM L-cysteine dissolved in TES-SB buffer (20 mM Tris-base pH 8.0, 1 mM EDTA, 250 mM sucrose, 2 mM L-serine, and 20 mM boric acid), and samples were incubated for 20 min at 37 °C. The reaction was stopped via the addition of 50 µL of 200 mM sulfosalicylic acid, followed by incubation at 4 °C for 20 min. Samples were centrifuged at 1300× *g* for 5 min at 4 °C, and 20 µL of supernatant was transferred to a 96-well plate. Then, 180 µL of 10 mM NDA was added to the supernatants, followed by incubation for 15 min in darkness at room temperature to allow for the formation of the NDA-Glu-Cys complex (NDA-γ-GC). The fluorescence intensity of the NDA-γ-GC complex was measured at λ = 485 nm for excitation and λ = 535 nm for emission in a fluorescence plate reader (Synergy HT, Biotek, Winooski, VT, USA). In parallel, each sample was treated to remove the basal GSH levels. In these tubes, L-cysteine was added after sulfosalicylic acid. Fluorescence levels were compared with a GSH standard curve, and GCL activity was expressed as µg of NDA-γ-GC per mg of protein.

GPx activity was measured using a previously described spectrophotometric method [36]. The reaction mixture consisted of 50 mM phosphate buffer pH 7.0, 1 mM EDTA, 1 mM sodium azide (catalase inhibitor), 0.2 mM NADPH, 1 U/mL of GR, and 1 mM GSH. One hundred microliters of the homogenates was added to 0.8 mL of reaction mixture and incubated for 5 min at room temperature. The reaction was initiated by the addition of 0.1 mL of 0.25 mM H_2_O_2_ solution. Absorbance at 340 nm was recorded for 3 min every 30 s, and the activity was calculated from the slope of these lines (μmol of NADPH oxidized per min) using the molar extinction coefficient of NADPH (6.22 × 10^3^ M^−1^cm^−1^). Blank reactions with homogenates replaced by distilled water were subtracted from each assay. GPx activity was expressed as U/mg protein.

GR activity was measured using a previously described spectrophotometric method [34]. The reaction mixture consisted of 100 mM phosphate buffer pH 7.6, 1 mM EDTA, 1 mM GSSG, and 1 mM NADPH. One hundred microliters of homogenates were added to 0.9 mL of the reaction mixture. Absorbance at 340 nm was recorded for 2 min every 15 s, and the activity was calculated from the slope of these lines (μmol of NADPH oxidized per min) using the molar extinction coefficient of NADPH (6.22 × 10^3^ M^−1^cm^−1^). GR activity was expressed as U/mg protein.

GST activity was measured using a previously described spectrophotometric method [37]. The reaction mixture consisted of 0.5 mM phosphate buffer pH 6.5, 1 mM GSH and 1 mM CDNB. One hundred microliters of homogenates were added to 0.9 mL of the reaction mixture. Absorbance at 340 nm was recorded for 2 min every 15 s and the activity was calculated using the molar extinction coefficient of conjugate-oxidized GSH-2,4-dinitrobenzene (9.6 × 10^3^ M^−1^cm^−1^). GST activity was expressed as U/mg protein.

G6PDH activity was measured using a previously described spectrophotometric method [38]. The reaction mixture consisted of 55 mM Tris-HCl buffer pH 6.5 with 3.3 mM MgCl_2_, 100 mM glucose 6-phosphate, and 6 mM β-NADP^+^. Ten microliters of homogenates were added to 0.29 mL of the reaction mixture. NADPH formation reflected an increase of absorbance, which was recorded for 10 min every 1 min at 340 nm. Activity was calculated using the molar extinction coefficient of NADPH (6.22 × 10^3^ M^−1^cm^−1^). G6PDH activity was expressed as U/mg protein.

SOD activity was measured using the reduction of nitrobluetetrazolium (NBT) spectrophotometric method. One hundred and sixty-five microliters of samples were added to 815 µL of reaction mixture (122 µM xanthine, 122 µM EDTA, 30.6 µM NBT, 49 mM Na_2_CO_3_, and 0.006% BSA). The reaction was initiated via the addition of 20 µL of 0.46 U/mL xanthine oxidase (XO) and samples were incubated for 15 min at 27 °C. The reaction was stopped via the addition of 330 µL of 0.8 mM CuCl_2._ The absorbance was measured at 560 nm (Synergy HT, Biotek, Winooski, VT, USA). In parallel, the absorbance of an unspecific tube (165 µL of sample, 815 µL of reaction mix, and 20 µL of distilled water) was measured, and the value was subtracted from each assay sample. SOD activity was expressed as U/mg protein.

CAT activity was measured using a spectrophotometric method. Twenty-five microliters of samples were added to 725 µL of reaction mix (6 mM KH_2_PO_4_, 4 mM Na_2_HPO_4_ pH 7.0, and 30 mM H_2_O_2_), and the absorbance was measured immediately at 240 nm every 15 s for 3 min. The activity was calculated in the time period where the reaction was lineal. A blank reaction with distilled water replacing homogenates was subtracted from each assay. The activity was calculated from the slope of these lines following a first-order kinetic model. CAT activity was expressed as *k*/mg of protein.

### 2.10. GSH Levels

GSH levels were determined using a previously described fluorometric method [39]. Striatal samples were sonicated in 0.75 mL of EDTA-phosphate buffer pH 8.0 (0.1 M phosphates and 5 mM EDTA) plus 1 mL of 25% H_3_PO_4_. Homogenates were centrifuged at 3000× *g* for 15 min and supernatants were separated. Five hundred microliters of supernatant were added to 4.5 mL of EDTA-phosphate buffer, and then 100 µL of this mixture were added to 1.8 mL of EDTA-phosphate buffer plus 100 µL of 7.5 mM OPA. Samples were incubated at room temperature in darkness for 15 min and the fluorescence signals were recorded in a fluorescence plate reader (Synergy, Winooski, VT, USA) at 420 nm for emission and 350 nm for excitation. GSH levels were expressed as µg GSH per mg protein.

### 2.11. Statistical Analysis

**Figure 2:**
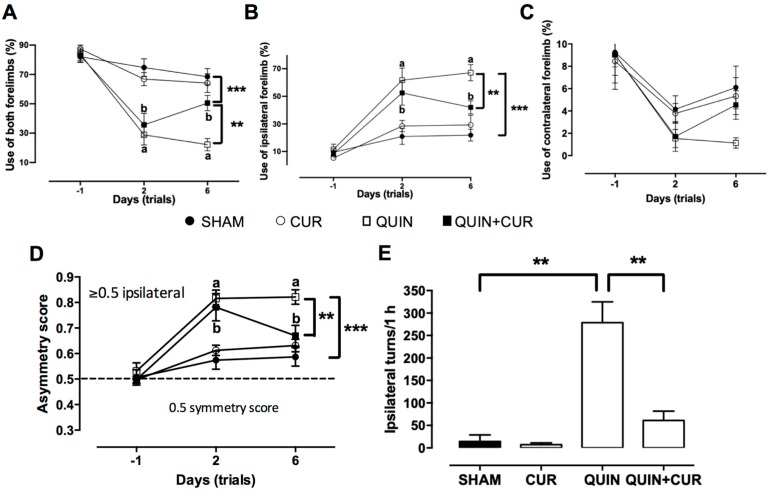
Effect of curcumin (CUR) on quinolinic acid (QUIN)-induced deficit motor skills. Motor evaluation was done on day −1 (one day before to striatal injection), and days 2 and 6 after QUIN injection; in (**A**) the percentage of use of both forelimbs, in (**B**) the percentage of use ipsilateral forelimb, in (**C**) the percentage of use contralateral forelimb are presented, and in (**D**) the asymmetry score. The rotation behavior test was evaluated at day 6 after QUIN injection; apomorphine (1 mg/kg, s.c.) was administered and the number of ipsilateral turns was quantified for 1 h (**E**). Data are expressed as mean ± SEM (*n* = 15). ** *p* < 0.01, and *** *p* < 0.001. ^a^ *p* < 0.001 vs. day −1, and ^b^ *p* < 0.001 vs. day −1. Panels A–D were analyzed using two-way ANOVA and panel E was analyzed using one-way ANOVA followed by a post hoc Tukey’s test.

Data were expressed as mean ± SEM. One-way analysis of variance (ANOVA) was used, followed by a post hoc Tukey’s test and the Figure 2A–D panels were analyzed using two-way ANOVA followed by a post hoc Tukey’s test using the Prism 6.0 software (Graph Pad, San Diego, CA, USA). Values of *p* < 0.05 were considered statistically significant.

## 3. Results

### 3.1. CUR Improved QUIN-Induced Motor and Histological Impairment

The data obtained in the motor assessment showed an increase in the use of both forelimbs (49.1%) after 6 days of treatment with CUR (QUIN+CUR group) compared to the QUIN group (25.25%) (Figure 2A). Moreover, QUIN significantly increased the use of the ipsilateral forelimb to the lesion side (64.77%) with respect to the SHAM group (21.89%, Figure 2B). Treatment with CUR (QUIN+CUR group) decreased the percentage (44.2%) of use of the ipsilateral forelimb to the lesion side at day 6 compared to QUIN group (Figure 2B). No significant changes were observed in the use of the contralateral forelimb to the lesion side at day 6 (Figure 2C).

Rats of all groups showed a symmetric exploration score at day −1 (use of both forelimbs) near to 0.5 (SHAM = 0.507, CUR = 0.498, QUIN = 0.531, and QUIN+CUR = 0.498). Rats that received QUIN (QUIN and QUIN+CUR groups) showed a significantly increased score (0.816 and 0.781, respectively) at day 2. Scores at day 6 in the QUIN+CUR group were decreased (0.67) with respect to the QUIN group (0.821) (Figure 2D). Scores in the SHAM and CUR groups did not significantly increase throughout the test (Figure 2D). Moreover, QUIN significantly increased the number of turns to the ipsilateral side (278.6 turns) with respect to the SHAM group (14.5 turns). Administration of CUR (QUIN+CUR) post-lesion significantly decreased the ipsilateral turns induced by QUIN (61 turns) (Figure 2E).

**Figure 3:**
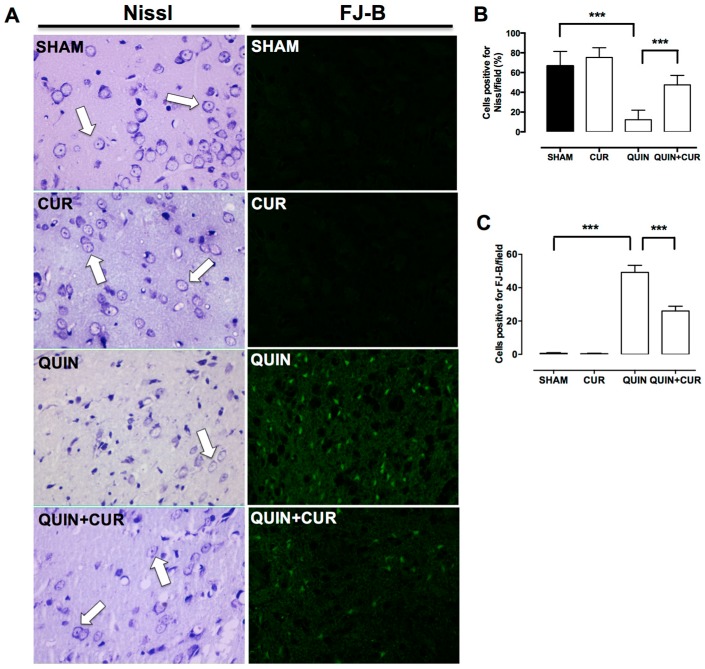
Effect of curcumin (CUR) on quinolinic acid (QUIN)-induced histological damage. Histological analysis was evaluated at day 7 after the QUIN injection. A representative photomicrograph (40×) of each group of the right striatum for (**A**) Nissl and FJ-B staining are presented. White arrows indicate cells positive for Nissl. Quantification of the percentage (%) of (**B**) cells positive for Nissl/field and (**C**) cells positive for FJ-B/field, counted in three randomly selected fields along the right striatum are presented. Data are expressed as mean ± SEM (n = 5 for FJ-B and n = 3 for Nissl staining). *** *p* < 0.001.

To evaluate the cellular damage induced by QUIN, Nissl staining was carried out. Cells positive for Nissl were observed in SHAM and CUR groups 7 days after the saline solution injection (Figure 3A,B). QUIN caused an important loss of cells positive for Nissl (12.2%) along the striatum respect to SHAM group (66.7%) (Figure 3A,B). CUR treatment (QUIN+CUR group) preserved cells positive for Nissl compared to the QUIN group (47.55%) (Figure 3A,B).

Moreover, cells in degeneration were evaluated through the FJ-B staining. QUIN significantly increased the number of cells positive to FJ-B (49.2%) in the right striatum with respect to SHAM group (0.6%) (Figure 3A,C). Daily administration of CUR (QUIN+CUR group) significantly decreased (26%) striatal degeneration induced by QUIN (Figure 3A,C).

### 3.2. CUR Increased BDNF, Phospho-ERK1/2, and Nrf2 Levels in the Striatum

**Figure 4:**
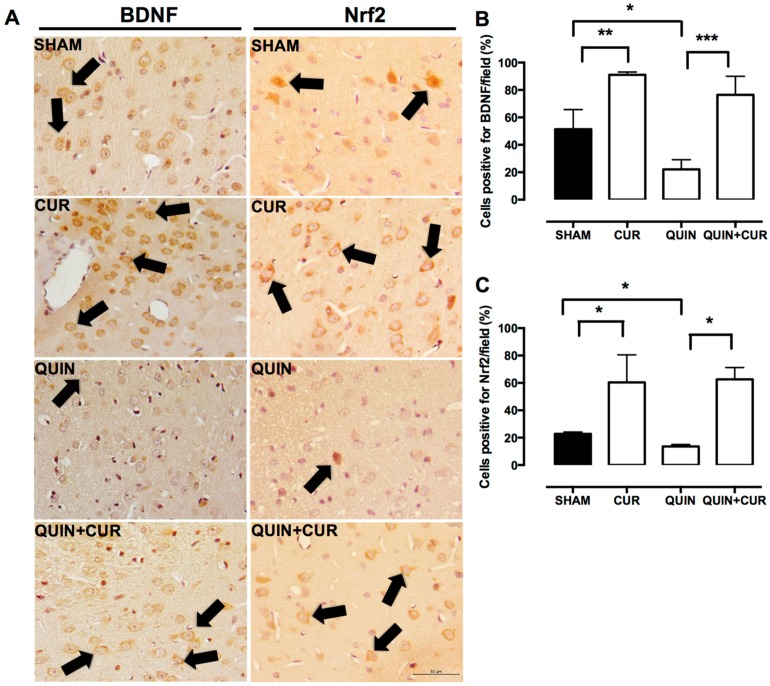
Effect of quinolinic acid (QUIN) and curcumin (CUR) on brain-derived neurotropic factor (BDNF) and nuclear factor erythroid 2-related factor 2 (Nrf2) levels. A representative image (40×) of immunohistochemistry assays of each group for the detection of BDNF and Nrf2 in the striatum are presented (**A**). Black arrows indicate cells positive for the respective protein. Graphs indicate the percentage of cells positive for (**B**) BDNF and (**C**) Nrf2, counted in three randomly selected fields along the right striatum. Data were expressed as mean ± SEM (n = 3). * *p* < 0.05, ** *p* < 0.01, and *** *p* < 0.001.

We observed an abundant expression of BDNF, mainly in the perinuclear region in the SHAM group. CUR alone increased the cells positive for BDNF compared with the SHAM group. In animals of the QUIN group, a decrease in BDNF levels was observed, compared to the SHAM and CUR groups. The CUR treatment (QUIN+CUR group) avoided the decrease in the number of BDNF- cells positive in the striatum (Figure 4A,B).

A modest number of cells positive for Nrf2 were observed in the striatum of the SHAM group. CUR alone increased the cells positive for Nrf2 compared with the SHAM group. In animals of the QUIN group, a decrease of the cells positive for Nrf2 was observed, compared to the SHAM group. The treatment with CUR (CUR+QUIN) increased the number of cells positive for Nrf2 in the striatum (Figure 4A,C).

**Figure 5:**
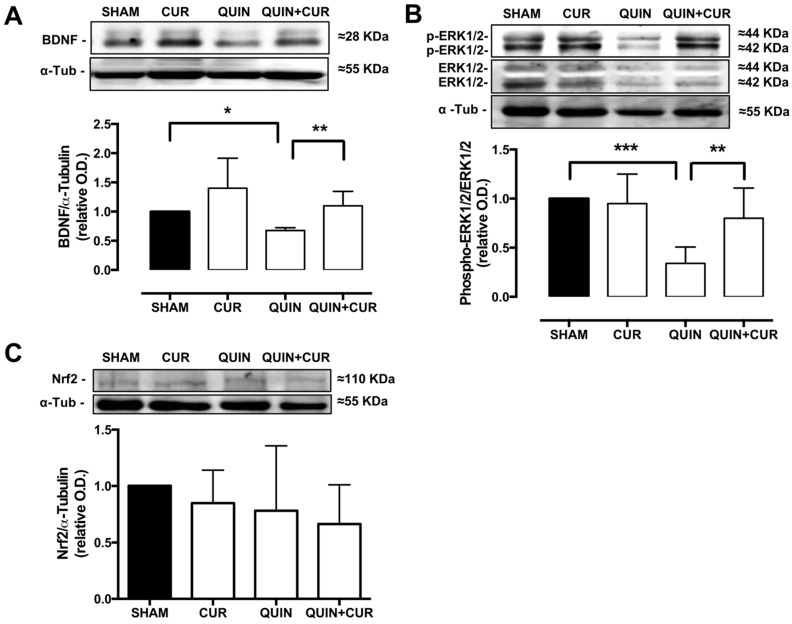
Effect of quinolinic acid (QUIN) and curcumin (CUR) on brain-derived neurotropic factor (BDNF), phosphorylated mitogen activated protein kinase (MAPK) protein extracellular signal-regulated kinase-1/2 (phospho-ERK1/2), and nuclear factor erythroid 2-related factor 2 (Nrf2) levels. Representative blots (upper panels) and quantification of the optical density (O.D.) ratio (lower panels) of (**A**) BDNF, (**B**) phospho-ERK1/2, and (**C**) total Nrf2 in the striatum are presented. Data are expressed as mean ± SEM (n = 3). * *p* < 0.05, ** *p* < 0.01, and *** *p* < 0.001.

To corroborate the changes observed in BDNF and Nrf2 levels using immunohistochemistry, Western blot analyses were performed. The QUIN significantly decreased the levels of BDNF (0.67) in the striatum, whereas the treatment with CUR (CUR+QUIN) restored the levels of BDNF (1.1) similar to the SHAM group (Figure 5A). No changes were observed in total Nrf2 levels (Figure 5C).

Finally, the phosphorylation of ERK1/2 was evaluated as a possible mechanism involved in the Nrf2 activation. QUIN decreased the phospho-ERK1/2 levels (0.34) in the striatum and the treatment with CUR restored the phospho-ERK1/2 levels (0.8) at 7 days after treatment (Figure 5B).

### 3.3. CUR Enhanced Cellular Antioxidant Capacity by Increasing Levels and Activity of Antioxidant Enzymes and GSH Levels

**Figure 6:**
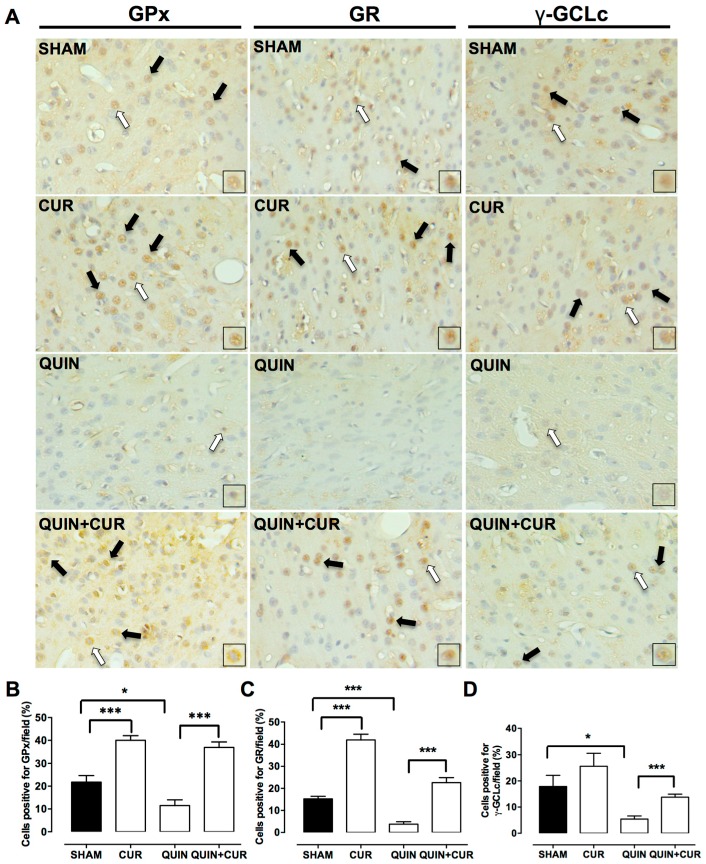
Effect of quinolinic acid (QUIN) and curcumin (CUR) on the protein levels of the enzyme regulated by the nuclear factor erythroid 2-related factor 2 (Nrf2). Immunohistochemistry assays were performed at day 7 after the quinolinic acid (QUIN) injection. Representative photomicrographs (40×) of cells positive for glutathione peroxidase (GPx), glutathione reductase (GR), and γ-glutamylcysteine ligase, catalytic subunit (γ-GCLc) in the striatum are presented (**A**). Black arrows indicate cells positive for each protein. Boxes represent the magnified positive cells indicated by white arrows. Graphs indicate the percentage of cells positive for (**B**) GPx, (**C**) GR, and (**D**) γ-GCLc, counted in three randomly selected fields along the right striatum. Data are expressed as mean ± SEM (n = 5). * *p* < 0.05 and *** *p* < 0.001.

The QUIN injection decreased the number of cells positive for GPx (11.5%, Figure 6A,B), GR (3.75%, Figure 6A,C), and γ-GCLc (5.41%, Figure 6A,D) compared to the SHAM group. The treatment with CUR (QUIN+CUR group) significantly increased the number of cells positive for GPx (37%, Figure 6A,B), GR (22.7%, Figure 6A,C), and γ-GCLc (13.8%, Figure 6A,D) with respect to the QUIN group on day 7. Finally, daily administration of CUR alone significantly increased the number of cells positive for GPx (40%, Figure 6A,B), and GR (41.87%, Figure 6A,C) with respect to the SHAM group (21.8% and 15.27%, respectively).

**Figure 7:**
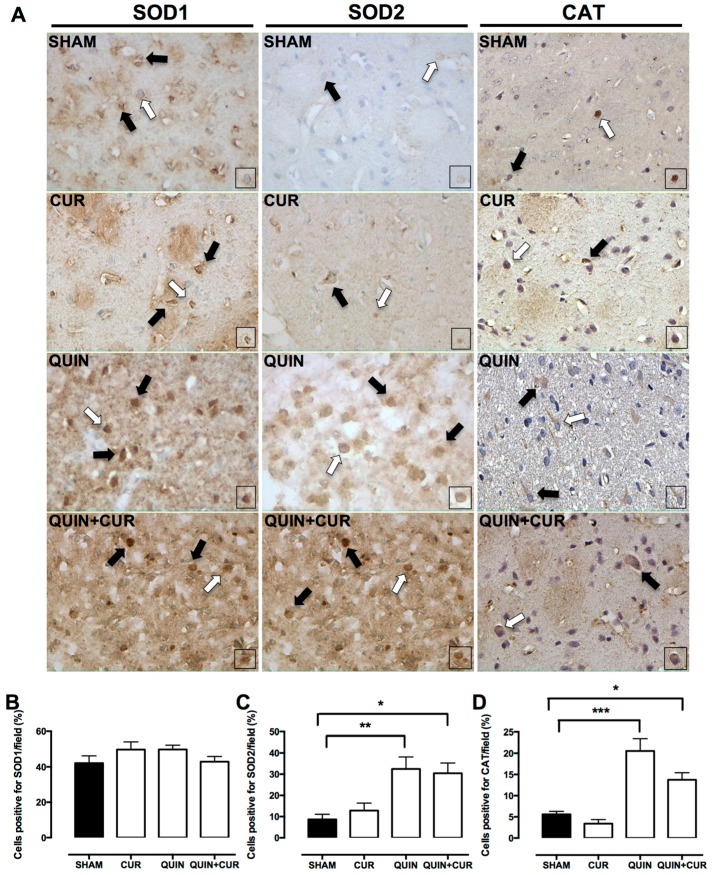
Effect of quinolinic acid (QUIN) and curcumin (CUR) on the protein levels of the enzyme regulated by the nuclear factor erythroid 2-related factor 2 (Nrf2). Immunohistochemistry assays were performed at day 7 after the quinolinic acid (QUIN) injection. Representative photomicrographs (40×) of cells positive for superoxide dismutase 1 (SOD1), superoxide dismutase 2 (SOD2), and catalase (CAT) in the striatum are presented (**A**). Black arrows indicate cells positive for each protein. Boxes represent the magnified cells positive indicated by white arrows. Graphs indicate the percentage of cells positive for (**B**) SOD1, (**C**) SOD2, and (**D**) CAT, counted in three randomly selected fields along the right striatum. Data are expressed as mean ± SEM (n = 3). * *p* < 0.05, ** *p* < 0.01, and *** *p* < 0.001.

QUIN and CUR treatment did not change the SOD 1 levels (Figure 7A,B). A scarce number of cells positive for SOD2 and CAT were observed in the SHAM and CUR groups (Figure 7A). However, QUIN injection increased SOD2 (31%, Figure 7A,C) and CAT (20%, Figure 7A,D) levels in the striatum, which did not change using the CUR treatment (QUIN+CUR group: SOD2 29% and CAT 15%).

**Figure 8:**
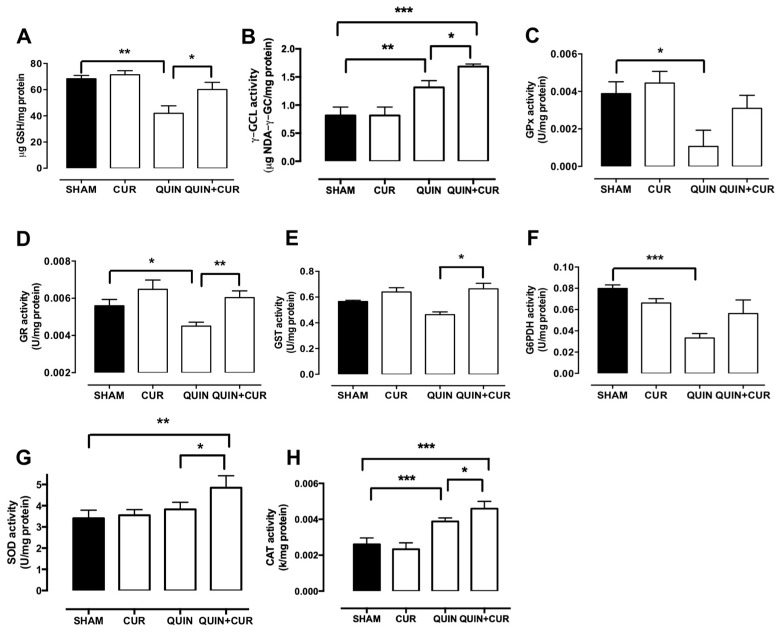
Effect of quinolinic acid (QUIN) and curcumin (CUR) on the glutathione (GSH) levels and enzymatic activity of proteins regulated by nuclear factor erythroid 2-related factor 2 (Nrf2). Assays were performed at 7 days after the QUIN injection. GSH levels (**A**) and enzyme activity of (**B**) γ-glutamylcysteine ligase (γ-GCL), (**C**) glutathione peroxidase (GPx), (**D**) glutathione reductase (GR), (**E**) glutathione S-transferase (GST), (**F**) glucose 6 phosphate dehydrogenase (G6PDH), (**G**) superoxide dismutase (SOD), and (**H**) catalase (CAT) in the striatum are presented. Data are expressed as mean ± SEM (n = 5 for GPx, GR, GST, G6PDH, and GSH and n = 4 for γ-GCL, SOD, and CAT). * *p* < 0.05, ** *p* < 0.01, and *** *p* < 0.001.

QUIN treatment significantly decreased the GSH levels in the right striatum (41.95 µg GSH/mg protein) on day 7 compared to the SHAM group (68.3 µg GSH/mg protein). CUR treatment (QUIN+CUR group) avoided the QUIN-induced decrease of GSH levels (60.12 µg GSH/mg protein) (Figure 8A). The GSH levels were unchanged in the CUR group.

QUIN injection significantly decreased the activity of GPx (0.0010 U/mg protein, Figure 8C), GR (0.0045 U/mg protein, Figure 8D), and G6PDH (0.033 U/mg protein, Figure 8F) with respect to the SHAM group (GPx: 0.0038 U/mg protein, GR: 0.0056 U/mg protein, and G6PDH: 0.079 U/mg protein, Figure 8C,D,F, respectively). Treatment with CUR (QUIN + CUR group) only prevented the decline of the enzyme activity of GR (0.006 U/mg protein, Figure 8D). QUIN treatment was unable to decrease GST activity (Figure 8E); interestingly, GST activity in the QUIN+CUR group was higher than in the QUIN group (Figure 8E). Surprisingly, QUIN administration increased the γ-GCLc and CAT activity, but the major increase was observed in the QUIN+CUR group (Figure 8B,H). No changes were observed in the SOD activity (Figure 8G).

## 4. Discussion

In the present study, we demonstrated that the post-lesion treatment with CUR confers protection against the cellular damage, neuronal degeneration, and motor alterations induced by QUIN. This protective effect could be associated with the ability of CUR to increase Nrf2 levels. The CUR treatment also increased the phospho-ERK1/2 and BDNF levels at 7 days after QUIN injection, suggesting that the mechanism by which CUR increases Nrf2 levels involved the modulation of BDNF levels and ERK1/2 activation. Supporting our data, there is evidence indicating that the post-treatment with CUR increases the Nrf2 levels in rats subjected to middle cerebral artery occlusion (MCAO) [40], and in rat cortical neurons culture submitted to oxygen-glucose deprivation (OGD) [41]. In addition, Ishii et al. [42] proposed that BDNF plays a key role in the regulation of Nrf2 in astrocytes and the metabolic cooperation between astrocytes and neurons. Astrocytes predominantly express truncated receptor TrkBr (TrkB.T1), whose activation leads to the protein kinase C ζ (PKCζ)-dependent Nrf2 stabilization [42]. Moreover, it was reported that BNDF activates the ERK1/2 pathway, inducing the Nrf2-mediate antioxidant response in a model of traumatic brain injury with transplantation of neuronal stem cells [43]. Also, Bucolo et al. [24] reported that CUR provides protection in retinal pigment cells exposed to a high glucose insult through the activation Nrf2/heme oxygenase 1 signaling, which involves the ERK pathway. However, the possibility that CUR may be acting on some cysteine residues of Keap1, the repressor protein of Nrf2, cannot be discarded [44].

We observed that CUR treatment avoided the decrease of the protein levels regulated by Nrf2 GPx, GR, and γ-GCLc induced by QUIN. However, CUR was able to prevent the decrease only in the GR activity. Supporting our results, there is evidence indicating that the CUR increases both protein expression and enzyme activity of thioredoxin in cells at 24 h after reoxygenation in a model of OGD [45]. Also, an increase in the expression of mRNA and protein levels of heme oxygenase 1 was observed when CUR was injected 15 min after MCAO [40]. We found that QUIN injection increased the SOD2 and CAT levels, but only increased the CAT activity. Meanwhile, in the QUIN+CUR group, a major increase was observed in SOD and CAT activities. Similar to these results, another study reported an increase in superoxide dismutase activity and NADPH quinone oxidoreductase 1 protein levels in response to treatment with CUR (300 mg/kg) 1 h after MCAO [45]. This suggests that the activation of proteins regulated by Nrf2 are related to the neuroprotective effect of CUR.

Also, we observed an increase in the GSH content in the striatum after CUR treatment at 7 days post-injury with QUIN. Disturbances in GSH homeostasis are implicated in the etiology and progression of numerous conditions, including cancer, inflammatory processes, and neurodegenerative diseases [46]. There are reports indicating that CUR increases the cellular content of GSH in cultures of astroglial cells [47]. Furthermore, an increase of GSH levels was observed in the cerebellum, striatum, hippocampus, and frontal cortex of rats treated with CUR (100 mg/kg, i.g.) and exposed to lead [48]. Optimal levels of GSH in the brain depend on: (a) its synthesis, (b) its regeneration, and (c) its consumption. The synthesis of GSH depends mainly on the activity of γ-GCL, which catalyzes the binding of glutamate to cysteine, the limiting step in the synthesis of GSH. In this study, we observed that QUIN injection decreased the γ-GCLc levels but increased the γ-GCL activity, and the CUR treatment prevented the decrease of γ-GCLc levels and increased the γ-GCL activity even more at day 7 after the QUIN administration. Lavoie et al. [49] reported that CUR led to an increase in γ-GCL activity in neurons and astrocytes, an effect associated with an increase in the GSH content. However, the biological meaning of the increase in γ-GCL activity in the QUIN model must be studied.

Nr2 controls the expression of the enzymes responsible for regenerating GSH levels, such as GR, which keeps an optimal balance between its reduced (GSH) and oxidized form (GSSG), in response to cellular redox demand [10]. The observed increase in GR activity in response to post-treatment with CUR after a QUIN lesion may confer to the cell the ability to regenerate the appropriate amount of GSH once it has been oxidized. Similar results were reported in the hippocampus of rats subjected to subarachnoid hemorrhage, in which an increase in GR activity was found in response to treatment with CUR (100 mg/kg) for one week [50]. The reduction of GSSG, catalyzed by GR, consumes NADPH, so it is necessary to maintain optimal levels of this cofactor during the oxidizing event. In this study, a tendency to increase the G6PDH activity using CUR after a QUIN lesion was observed, which could be the result of providing adequate levels of NADPH. However, the NADPH used by GR may also be derived from the catalytic action of other enzymes, for example, from the malic enzyme that is also regulated by the Nrf2 [51]. The reaction catalyzed by GST uses GSH as the main substrate, so its activity is compromised and conditioned to optimal levels of GSH in the brain. The decrease in GST activity with the QUIN (not significant) could be a consequence of the decrease in the GSH levels in response to the QUIN. In contrast to our results, an increase in GST activity after infusion with QUIN (36.8 nmol) was reported in the cortex and hippocampus at 24 h, which was attributed to the ability of cells to detoxify lipoperoxidation products induced by the QUIN [52]; however, in our model, we used 240 nmol of QUIN and the GST activity was measured in the striatum, which could explain this discrepancy.

**Figure 9:**
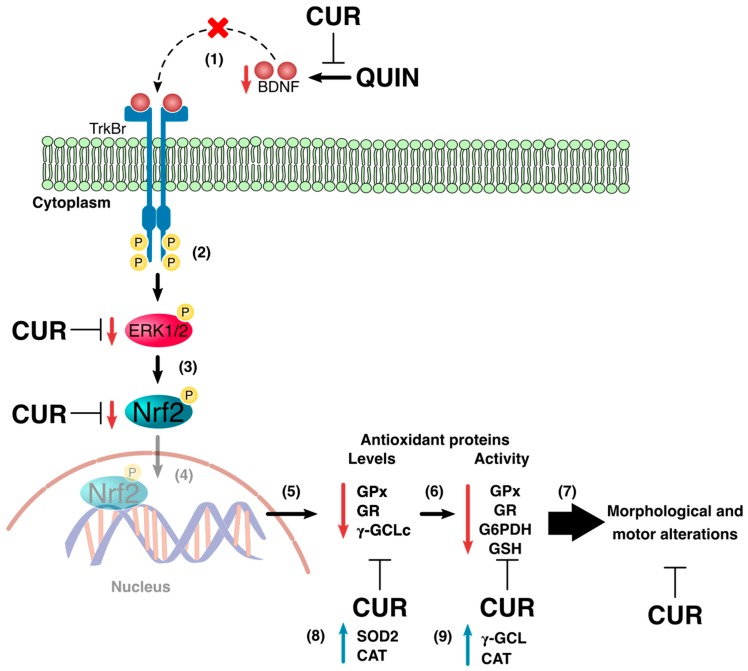
Curcumin (CUR) administration protected brain tissue against the damage induced by quinolinic acid (QUIN). QUIN administration decreased the protein levels of the brain-derived neurotropic factor (BDNF) on day 7, preventing the stimulation of its receptor the tropomyosin receptor kinase-B (TrkBr) (**1**). The decrease in TrkBr signaling avoided the protein extracellular signal-regulated kinase-1/2 (ERK1/2) activation (**2**) and consequently, the nuclear factor erythroid 2 related factor 2 (Nrf2) accumulated (**3**). This allowed for Nrf2 to be translocated into the nucleus (diffuse area) (**4**). The decrease in Nrf2 levels diminished the protein levels of glutathione peroxidase (GPx), glutathione reductase (GR) and γ-glutamylcysteine ligase catalytic subunit (γ-GCLc) (**5**), and the enzyme activity of GPx, GR, and glucose-6-phosphate dehydrogenase (G6PDH), as well as glutathione (GSH) levels (**6**), inducing cellular damage and motor alterations (**7**). Finally, QUIN injection increased superoxide dismutase 2 (SOD2) and CAT levels (**8**), and the γ-GCL and CAT activities (**9**). On the other hand, CUR treatment 24 h after QUIN injection prevented the decrease in BDNF levels, avoiding the reduction of ERK1/2 activation and Nrf2 stabilization and its nuclear translocation and activation, restoring the levels and the activity of antioxidant enzymes and GSH levels, improving morphological and motor alteration induced by QUIN.

In summary, the therapeutic effect of the CUR in the QUIN experimental model is associated with its ability to stabilize Nrf2 and regulate the expression of antioxidant enzymes through BDNF action on the activation of the ERK1/2. In addition, the increase in GSH levels in the striatum induced by CUR also could be part of the mechanism that blocks the neurotoxic effects induced by QUIN (Figure 9). However, it is possible that the reactive oxygen species induced by QUIN could also be responsible for the decrease of the Nrf2 and phospho-ERK1/2 levels. Finally, our data suggest that the regulation of Nrf2 activation could be a promising target for therapeutic intervention in neurodegenerative diseases.
